# A Rare Case of Lymphocytic Ascites

**DOI:** 10.7759/cureus.59760

**Published:** 2024-05-06

**Authors:** Thabuna Sivaprakasam, Mohammad Ali, Jaswanth Jasti, Randall Lamfers

**Affiliations:** 1 Internal Medicine, University of South Dakota Sanford School of Medicine, Sioux Falls, USA

**Keywords:** doxycycline, pelvic inflammatory disease, complex ascites, sexually transmitted infection (sti), chlamydia trachomatis, lymphocytic ascites

## Abstract

Ascites can manifest as a result of many conditions, with cirrhosis being the most common cause in the United States. Here, we present a case of lymphocytic ascites, a less common variant that occurred due to infection with Chlamydia trachomatis. This was a 37-year-old female with a history of substance and sexual abuse who presented with the chief complaints of abdominal pain, abdominal distension, and weight gain. She was febrile on admission with a distended, tender abdomen. The more common cardiac, renal, and hepatic causes were ruled out with extensive workup. Diagnosis and therapeutic paracentesis were done with fluid analysis significant for lymphocyte predominance and absence of malignant cells. Multi-modal imaging had ruled out suspicious malignant masses but CT abdomen/pelvis did show complex large volume ascites. Urine chlamydia and gonorrhea polymerase chain reaction (PCR) had resulted positive for chlamydia, leading us to start Doxycycline. Other infectious workups were negative, but ascitic fluid chlamydia NAAT was positive. Though initially worsening, the patient started showing significant clinical improvement after starting doxycycline, with the resolution of ascites and associated symptoms. This case report intends to bring to attention the importance of testing for chlamydia infection in cases of lymphocytic ascites, especially in sexually active females.

## Introduction

Ascites is defined as the pathological accumulation of fluid within the abdominal cavity. Though it is commonly attributed to hepatic, cardiac, or renal causes, infectious and malignant etiologies are also to be ruled out promptly. Lymphocytic ascites, defined by lymphocytic predominance in the ascitic fluid, is uncommon and evokes the investigation for rarer causes. It has been reported in many cases of cirrhotic ascites, tuberculous ascites, malignant lymphoma, and chronic lymphocytic leukemia [1]. It is also seen, but only rarely, in cases of Chlamydia (C.) trachomatis infection and pelvic inflammatory disease (PID). Our patient was one of such rarer presentations of chlamydia-related PID.

Peritoneal infection by chlamydia leading to PID presenting as ascites was described as early as 1980 in a 21-year-old female who presented with recurrent abdominal pain after an appendectomy [2]. But in the absence of accurate testing, such as polymerase chain reaction (PCR), a presumptive diagnosis of chlamydia infection was made and treatment with tetracyclines produced an excellent clinical response. Advancements in PCR testing have helped establish the causal relationship between the organism and ascites in patients such as ours. Pelvic inflammatory disease is caused by ascending infection from the lower to upper genital tract, including the endometrium, fallopian tubes, or adjacent structures. Peritonitis is a dreaded complication of PID, where organisms might spread from the genital tract to the adjoining peritoneum, causing inflammation. This course of spread would explain the peritonitis and the resulting ascites in our patient. Fitz Hugh Curtis syndrome is a rare complication of PID and is defined as an inflammation of the liver capsule, without the involvement of the liver parenchyma, with adhesion formation [3]. It was first described by Carlos Stajano in 1920 in Uruguay, and in 1930, Thomas Fitz-Hugh and Arthur Curtis connected the clinical features of abdominal pain with the classic “violin-string” adhesions [4]. With Neisseria gonorrhoeae assumed to be the sole cause, it was only in 1978 that Chlamydia trachomatis was also attributed to the syndrome [4]. The major difference between a patient presenting with this syndrome and just peritonitis with ascites as in our case, would be the presence of perihepatitis and adhesions, which contributes to the classic right upper quadrant abdominal pain of Fitz Hugh Curtis syndrome. Whereas, in solely Chlamydia-induced ascites, abdominal distension along with generalized pain would be the primary presentation. But, interestingly, cases have been described in the literature with this syndrome presenting as diffuse peritonitis with chronic ascites, without liver involvement [5]. Further reporting of cases and extensive review would be necessary to confirm such a variable clinical presentation of Fitz Hugh Curtis syndrome.

## Case presentation

**Figure 1:**
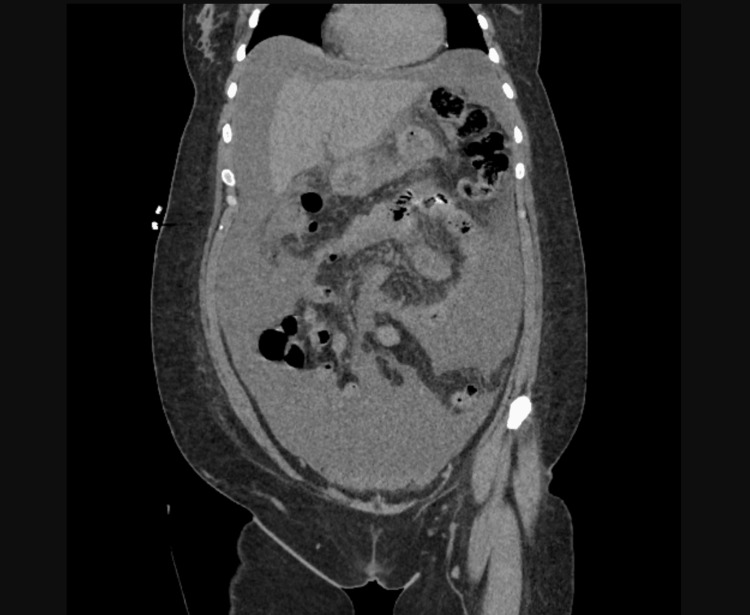
CT abdomen pelvis showing complex intra-peritoneal ascites

A 37-year-old female with a history of anxiety, depression, substance abuse, and sexual abuse presented with four days of chief complaints of generalized abdominal pain, abdominal distension, associated breathlessness, nausea, and vomiting. She also had pedal edema and a weight gain of 30 pounds within the past month. She denied a history of liver disease, renal disease, and heart disease. She reports that she was sexually assaulted a year ago followed by menorrhagia, which was ongoing, for which she did not seek treatment. Vital signs on presentation were significant for a temperature of 100.1 and tachycardia. Physical examination revealed a distended, tender abdomen with a positive fluid wave and dullness on percussion, and bilateral 1+ pedal edema. Bowel sounds were present. Labs showed a CBC with a hemoglobin of 7.5, mean corpuscular volume (MCV) of 56, WBC of 10.9, elevated absolute lymphocyte count, and an iron profile suggestive of iron deficiency anemia (Table 1). The chemistry and liver panel were unremarkable with a negative hepatitis panel. CT abdomen/pelvis revealed large volume abdominal pelvic ascites, possibly complex in nature due to suspected peritoneal enhancement concerning for peritonitis or peritoneal carcinomatosis (Figure 1).

**Table 1:** Complete blood count and differential

Test	Observed Value	Reference Range
RBC (M/uL)	4.85	3.80 - 5.30
Hemoglobin (g/dL)	7.4	11.5 – 15.8
Hematocrit (%)	28.4	35 - 45
MCV (fL)	58.6	80.0 – 98.0
WBC (K/uL)	10.9	4.0 – 11.0
Platelet count (K/uL)	877	140 - 400
Segmented neutrophils (K/uL)	5.6	1.8 – 8.0
Absolute eosinophils (K/uL)	0.0	0.0 – 0.7
Absolute lymphocytes (K/uL)	4.4	0.8 – 4.1
Absolute immature granulocytes (K/uL)	0.08	0.00 – 0.06

The presentation was initially concerning for peritonitis or malignancy with reduced suspicion of heart failure, nephrotic syndrome, or cirrhosis. Ceftriaxone and metronidazole were started empirically and the patient was hospitalized for management of pain and evaluation of ascites of unclear etiology. She underwent paracentesis with the removal of 4.6 liters of clear, yellow fluid. Fluid analysis showed 1616 nucleated cells with 64% lymphocytes, protein 4.6, glucose 60, lactate dehydrogenase (LDH) 907, and serum ascites albumin gradient (SAAG) of 1.3 (Table 2). Fluid cytology was negative for malignancy. A transvaginal ultrasound was done to further evaluate pelvic organs, which was not suspicious for any masses. Urine chlamydia and gonorrhea PCR came back positive for chlamydia, and IV doxycycline was added to the regimen.

**Table 2:** Peritoneal fluid analysis LDH: lactate dehydrogenase; SAAG: serum ascites albumin gradient

Ascitic fluid labs	On admission	3 days later
RBC	<2000	<2000
Nucleated cells	1,616	1,263
% Neutrophils	38	25
% Lymphocytes	49	64
% Mononuclear cells	13	11
Albumin (g/dL)	2.2	1.9
Amylase (U/L)	30	30
Glucose (mg/dL)	60	60
LDH (U/L)	709	907
Protein (g/dL)	5.10	4.6
SAAG	1.3	<1.1

The patient continued to have persistent fevers with increasing abdominal pain and distension during hospitalization. Paracentesis was repeated with 1.5 L of fluid removed and fluid analysis again showed 1263 nucleated cells, 64% lymphocytes, SAAG < 1.1, and similar protein and LDH, and cytology showed reactive lymphocytes.

Given the negative malignancy workup and lymphocyte-predominant ascites, further infectious investigations were pursued. Blood cultures, HIV serology, ascitic fluid cultures, fluid AFB smears, and cultures were negative. General surgery was consulted for consideration of diagnostic laparoscopy and omental biopsy if a comprehensive workup was negative. But the ascitic fluid chlamydia nucleic acid amplification test (NAAT) turned out to be positive, and the patient started showing clinical improvement after starting doxycycline. This confirmed the diagnosis of secondary bacterial peritonitis secondary to Chlamydia trachomatis infection. Infectious disease was consulted and the antibiotic regimen was switched to oral doxycycline and metronidazole for an additional 14 days. The patient had symptomatic improvement with the resolution of ascites and was discharged after being afebrile for more than 48 hours.

## Discussion

Pelvic inflammatory disease (PID) is an infection of the female upper genital tract that may include the endometrium, fallopian tubes, ovaries, or pelvic peritoneum [6,7]. Sexually transmitted organisms Chlamydia trachomatis and Neisseria gonorrhoeae have been implicated in a third to half of PID cases, even though it is often a polymicrobial infection [6,8]. PID is typically acute, but a more indolent presentation can occur, encompassing a wide spectrum of clinical presentations and may include any combination of endometritis, salpingitis, oophoritis, peritonitis, perihepatitis, ectopic pregnancy, chronic pelvic pain, tubo-ovarian abscess, and even infertility [6,7]. Sexually active adolescent and young adult women are the most affected, but many infections go undiagnosed and untreated [6,9]. Young age, multiple sexual partners, a history of sexually transmitted diseases (STDs) in the patient or their partner, inconsistent condom use, vaginal douching, smoking, drug, and alcohol abuse, and even IUD insertion are all associated with an increased risk of PID [7,10]. According to the National Health and Nutrition Examination Survey (NHANES) conducted in 2013 and 2014, the prevalence of a self-reported lifetime PID diagnosis was 4.4% among sexually experienced reproductive-aged women, equating to 2.5 million PID cases in women aged 18-44 years nationwide, implicating that clinicians follow chlamydia and gonorrhea screening recommendations for women to decrease the incidence of PID [11]. Chlamydia trachomatis is a sexually transmitted gram-negative bacterium that causes infection worldwide. In 2020, a total of 1,579,885 cases of Chlamydia trachomatis infection were reported to the Centers for Disease Control and Prevention (CDC), making it the most common notifiable sexually transmitted infection in the United States for that year [12]. Even though most infected males and females are asymptomatic, the pathogen is a significant cause of several common clinical syndromes as well as syndromes specific to different sexes such as pelvic inflammatory disease (PID) in females. PID secondary to chlamydia can manifest as endometritis, salpingitis, oophoritis, pelvic peritonitis or ascites, and perihepatitis (Fitz-Hugh-Curtis syndrome) [6,7].

The first description of ascites caused by C. trachomatis was by Muller-Schoop et al. in 1978 [13]. There were 11 patients suspected to have peritonitis, out of which nine patients had serological positivity for Chlamydia trachomatis and six of them had culture-negative ascites. Wolner-Hanssen et al isolated C. trachomatis from an infected patient's liver capsule in 1982, confirming the association [14]. Since then, several authors have reported cases of complicated chlamydial infections causing ascites with or without perihepatitis in multiple contexts [5,15-20].

Abdominal paracentesis is crucial to identify the cause of a patient's ascites, such as cirrhosis, malignancy, or heart failure, in addition to routine laboratory testing and imaging. For all patients with newly developed ascites, a diagnostic paracentesis is recommended which helps to determine the SAAG. SAAG is highly useful to classify the nature of ascites, and SAAG ≥ 1.1 g/dL predicts that the patient has portal hypertension with 97% accuracy [21]. A gradient of <1.1 g/dL indicates portal hypertension is less likely. Low ascitic SAAG values can be caused by multiple conditions, such as pancreatic and renal disease and peritoneal malignancy, and infections such as tuberculosis, hypothyroidism, or paraproteinemia [22]. The total protein concentration will further help narrow the differential diagnosis of ascites. Other ascitic fluid tests, including cultures, cell count and differential, gram stain, cytology, and tests for tuberculous peritonitis should be ordered in the appropriate setting. The ascitic fluid analysis in Chlamydia trachomatis infection is characterized by a low SAAG gradient and high protein with lymphocytic predominance, which is similar to other peritoneal pathologies [17,19,23]. There have been cases of lymphocytic ascites that were mistaken for peritoneal or ovarian tumors in the past [18,19,24,25], which in some cases, resulted in invasive testing. In 2017, Brun et al. presented a case report titled "Erroneously Suspected Ovarian Cancer in a 38-Year-Old Woman with Pelvic Inflammatory Disease and Chlamydia." They noted the importance of carefully reviewing every ovarian cancer diagnosis, particularly in young patients, even when the presentation appears typical with enhanced peritoneum on radiology and elevated cancer antigen on cytology [19]. There are also some reports of high adenosine deaminase levels in chlamydia infections similar to peritoneal tuberculosis [17].

The current recommendations from the CDC advise that all sexually active women under the age of 25 should undergo screening to avert potential infectious complications [26]. Due to the rarity of Chlamydial ascites, there are no formal recommendations for performing routine testing in the workup of ascites. A combination of ascitic fluid analysis, including cell count, culture, and cytology; cervical swab cultures; laparoscopy with biopsies; and NAAT of both the cervical swabs and ascitic fluid can help confirm any diagnosis prior to a laparotomy.

The treatment of choice for C. trachomatis-associated ascites is a course of azithromycin or doxycycline. Oral doxycycline 100 mg twice daily for a duration of 10-14 days has been widely used and recommended for previous cases [6,13,16,19,20]. CDC recommends IV doxycycline 100 mg every 12 hours as part of an appropriate combination regimen, and transition to oral therapy after 24 to 48 hours of sustained clinical improvement to complete a 14-day total course in severe pelvic inflammatory disease [6]. A literature review demonstrates that antibiotics lead to improvement in the symptoms caused by infections including ascites [16,27,28]. Some studies suggest that ascites caused by Chlamydia infection could resolve on its own and might not always require antibiotic treatment [29,30].

## Conclusions

Chlamydia trachomatis is commonly implicated in many infectious presentations. It is more commonly described as the causative agent in conditions like cervicitis, vaginitis, urethritis, prostatitis, proctitis, pelvic inflammatory disease, and reactive arthritis. Nevertheless, it is crucial to recognize the less common presentations too, in conjunction with the clinical presentation. Though previously observed occasionally, it is challenging to promptly include the diagnosis of Chlamydia-induced peritonitis in the setting of more common possibilities of ascites. With the availability of reliable testing and effective antibiotics, early suspicion and diagnosis would greatly benefit the patient not only symptomatically but also help prevent the long-term complications of PID, including chronic pelvic pain, infertility, ectopic pregnancy, and tubo-ovarian abscess.
